# Temporal and Spatial Predictability of an Irrelevant Event Differently Affect Detection and Memory of Items in a Visual Sequence

**DOI:** 10.3389/fpsyg.2016.00065

**Published:** 2016-02-02

**Authors:** Junji Ohyama, Katsumi Watanabe

**Affiliations:** ^1^Department of Information Technology and Human Factors, National Institute of Advanced Industrial Science and TechnologyTsukuba, Japan; ^2^Department of Intermedia Art and Science, Waseda UniversityTokyo, Japan; ^3^Research Center for Advanced Science and Technology, The University of TokyoTokyo, Japan

**Keywords:** event, spatial predictability, temporal predictability, detection, working memory, visual short-term memory, recognition, attention

## Abstract

We examined how the temporal and spatial predictability of a task-irrelevant visual event affects the detection and memory of a visual item embedded in a continuously changing sequence. Participants observed 11 sequentially presented letters, during which a task-irrelevant visual event was either present or absent. Predictabilities of spatial location and temporal position of the event were controlled in 2 × 2 conditions. In the spatially predictable conditions, the event occurred at the same location within the stimulus sequence or at another location, while, in the spatially unpredictable conditions, it occurred at random locations. In the temporally predictable conditions, the event timing was fixed relative to the order of the letters, while in the temporally unpredictable condition; it could not be predicted from the letter order. Participants performed a working memory task and a target detection reaction time (RT) task. Memory accuracy was higher for a letter simultaneously presented at the same location as the event in the temporally unpredictable conditions, irrespective of the spatial predictability of the event. On the other hand, the detection RTs were only faster for a letter simultaneously presented at the same location as the event when the event was both temporally and spatially predictable. Thus, to facilitate ongoing detection processes, an event must be predictable both in space and time, while memory processes are enhanced by temporally unpredictable (i.e., surprising) events. Evidently, temporal predictability has differential effects on detection and memory of a visual item embedded in a sequence of images.

## Introduction

In our daily lives, countless visual changes around us occur in parallel; however, we only perceive or memorize some of these as salient events, and later recall fragmented event scenes that are abstracted from the important parts of ever-changing sequential information. Attention plays an important role in identifying relevant spatio-temporal changes, and these salient changes might be perceived as an event. However, it has not yet been examined what types of visual changes attract attention and are selectively remembered as salient events, or how the constantly changing sequence of information is influenced by the events. In the present study, we examined the effect of a task-irrelevant event on both detection and memory of time sequence information.

The effects of a visual event or cue on memory have been widely examined in studies of visual short-term memory and its capacity (Luck and Vogel, 1997; Chun and Jiang, 1998; Wolfe et al., 2006). Schmidt et al. (2002) reported that a visual change that was spatially congruent or incongruent in relation to the location of an array of items had a cueing effect on memory. Furthermore, they suggested that visual cues enhance memory of an item presented at the location of a cue. Hollingworth (2003) reported that the recognition memory of a cued item was more accurate than that of other items encountered in daily situations. However, in some conditions examined in other exogenous cueing studies, the cued advantage for detection or memory accuracy was less effective (Hauer and MacLeod, 2006; Santangelo et al., 2008). The difference between effective and ineffective results might depend on the saliency or predictability of the cue. Moreover, previous studies have reported that salient visual change in context increases attention and influences later memory process of the events (Newtson and Engquist, 1976; Fabiani and Donchin, 1995; Hunt, 1995; Ranganath and Rainer, 2003; Swallow et al., 2009).

Ohyama and Watanabe (2010) have reported that task-irrelevant visual change events affect recognition memory of a sequence of items, and showed that the memory enhancement effect occurred only for recognition memory accuracy of an item that simultaneously appeared with the task-irrelevant event in a sequence of items. Ohyama and Watanabe (2010) kept the spatio-temporal predictability of the event the same in all experiments; specifically, the event timing was unpredictable, and the event location was predictable. However, whether temporal unpredictability is important, spatial predictability is important, both are important and related, or neither is important has not been confirmed. For example, there was a possibility that the object-based effect in the previous study was peculiar to the spatially predictable event condition. In that case, if the event location was unpredictable, the unexpected event at a different location from the item sequence would attract more attention than the predictable event, therefore, the memory enhancement effect would occur in spatially unpredictable event at different location. Moreover, there was a possibility that the spatially predictable event in previous study was implicitly regarded as task-related event and affected memory. In that case, the events in both spatially and temporally unpredictable conditions would be easily ignored as task-irrelevant events; therefore, the memory enhancement effect would be decreased in spatially unpredictable condition. In another case, there was a possibility that event occurrence was only an important factor for the memory enhancement effect regardless of spatio-temporal predictability. Therefore, it remains unclear if the spatio-temporal predictability of an event is a critical factor that induces the memory enhancement effect. To examine the main effect of the memory enhancement effect found in spatially predictable and temporally unpredictable event, we prepared the counter part conditions that spatially unpredictable event and temporally predictable event condition, in the present study. Spatial predictability was controlled by fixed or randomized location of event selected from two possible locations related to the item sequence (i.e., at the same location within the stimulus sequence or at another location). Temporal predictability was controlled by fixed or randomized timing of event selected from three possible timings related to the item sequence.

A similar approach was used in studies of the subjective time expansion effect. In those studies, the researchers modified the predictability of events and discussed the relationship between predictability and the subjective time expansion effect to clarify the mechanism of the effect. First, Tse et al. (2004) reported that the duration of a salient, compared to non-salient, event was perceived to be longer if the event was temporally unpredictable and spatially predictable. It is possible that the recognition memory was enhanced by subjective time expansion since the event that evoked the memory enhancement effect had the same predictability conditions. Further, Pariyadath and Eagleman (2007) explained that the temporal predictability of an event is an essential factor for subjective time perception change, supporting this statement by comparing empirically predictable and unpredictable conditions for a sequence. Moreover, New and Scholl (2009) reported that a temporally and spatially unpredictable event drove subjective time dilation of an item that is at a different position from the event. Since spatial predictability was not related to the effect, they explained that the unpredictable event increased arousal and evoked the subjective dilation of time perception of the entire visual field. Similarly, if the memory enhancement effect is also found when the timing and location is unpredictable but not found when the timing or location is predictable, this memory enhancement might be explained as an after-effect of subjective time expansion. In other words, it may occur by the same arousal-driven process, along with subjective time expansion.

The relationship between memory enhancement and time perception effects can be established by investigating whether or not temporal and spatial predictabilities act as critical factors in the memory enhancement effect. Furthermore, it is possible to clarify the memory enhancement effect mechanism. Therefore, one of the purposes of the present study was to examine the relationship between the spatio-temporal predictability of an event and the event saliencies that drive the memory enhancement effect.

Another purpose of the present study was to show how the memory enhancement effect is related to the bottom–up attention, arousal, and the early perception process (i.e., detection or discrimination). Many previous studies have reported that perception is improved by salient visual events (Posner, 1980; Prinzmetal et al., 1986; Egly et al., 1994; Moore et al., 1998; Aston-Jones and Cohen, 2005; Zacks et al., 2007); therefore, it is possible that a task-irrelevant event affects the early perception process and that this perceptual effect affects later memory processing and improves recognition memory accuracy as an after-effect of the change in perceptual process. We also aimed to confirm if detection reaction times (RTs) of items in a serial presentation are affected by an event, using the stimuli conditions that were found to induce the memory enhancement effect (i.e., a spatially predictable and temporally unpredictable event that occurred at the same location of the item sequence) in a previous study (Ohyama and Watanabe, 2010). However, no event-related effect was found in the detection RT, even when the stimuli conditions were the same. This lack of a reduction in the detection RT in all experimental conditions might be related not to the event conditions, but rather to the task design of the detection experiment used in the previous studies. In other words, it is possible that an event that affects the detection process cannot be measured by the condition of the task or apparatus. However, if the task and apparatus were not the problem, there are two other hypotheses that may explain these negative results with regard to the detection RT. One hypothesis is that the event drove arousal and reduced the item detection RT, while, at the same time, the event attracted attention, reduced attention on the item as a distractor, and delayed the item detection RT, meaning that these plus and minus effects were offset and not seen in Ohyama and Watanabe’s (2010) study. Another hypothesis is that the event did not enhance detection in the perception process, instead only enhancing the memory process. To clarify these possibilities, both working memory and detection tasks were conducted for all experimental conditions, concerning temporal and spatial predictability in the present study.

In our previous study, the events were spatially predictable and temporally unpredictable, and under these conditions, we did not observe an effect on the detection RTs (Ohyama and Watanabe, 2010). However, previous studies have reported that detection RTs were shortened when the target presentation timing was predictable, compared to unpredictable (Correa et al., 2004, 2005). Therefore, it is possible that detection RTs are reduced when the event timing is predictable, such that an improvement in detection RTs would be related to the automatic facilitation of arousal or bottom–up attention. If the item perception process is influenced automatically by the event in the detection task, then the event must similarly affect the ongoing perception process during serial item presentation in the working memory task, because the event is task irrelevant.

If a reduction in detection RT occurs in a particular spatio-temporal predictability condition, and if the memory enhancement effect of that condition is more significant than in conditions where a reduction in detection RT was not found, then the saliency of item perception and arousal would increase in the early stages of item perception. Therefore, an improvement in memory for an item can be explained by facilitation of the early perceptual process. In contrast, if event predictability affects detection RT and recognition memory in different conditions, it is a more reasonable explanation that the event affects item memory in the later part of the memory process that is formed after the perception process.

In the present study, we examined whether the temporal and spatial predictability of a visual change event would affect detection and memory across 2 × 2 experimental conditions of spatio-temporal predictability. Therefore, we can clarify the critical conditions of a task-irrelevant event that enhance the memory of an item in sequential memory, and show what kind of perception or memory processing is related to the effect. We adopted similar stimuli and procedures to those used in our previous study (Ohyama and Watanabe, 2010), and examined the effect of both temporal and spatial predictability on the detection and memory of items embedded in a visual sequence. Specifically, the event was temporally and spatially predictable in Experiment 1, temporally unpredictable but spatially predictable in Experiment 2, temporally predictable but spatially unpredictable in Experiment 3, and temporally and spatially unpredictable in Experiment 4. In each experiment, we investigated the effect of a task-irrelevant event on the participant’s ability of both detection RT (in a target detection task) and recognition memory (in a working memory task).

## Materials and Methods

### Participants

Fifty-two participants from several universities who had normal or corrected-to-normal vision and were naïve to the purpose of the study participated in one of the four experiments. The gender and age of participants in each experiment are shown in **Table 1**. This study was approved by the ethics board of the University of Tokyo and all participants gave written informed consent.

**Table 1:** Temporal and spatial 2 × 2 event predictability design of the four experiments.

		**Event spatial location**	
			
		**Predictable**	**Unpredictable**
		(Two locations, consistent in all trials in both ipsi- and contra-lateral blocks)	(Two locations, ipsi- and contra-lateral events varied across trials)

			
**Event temporal position**	**Predictable** (One set timing, held consistent across all trials)	Experiment 1; *N* = 13 Age range (*M* = 21.2; *SD* = 1.99) Six males	Experiment 2; *N* = 13 Age range (*M* = 21.0; *SD* = 2.31) Seven males
			
	**Unpredictable** (Three different timings, varied across trials)	Experiment 3; *N* = 13 Age range (*M* = 21.5; *SD* = 1.27) Six males	Experiment 4; *N* = 13 Age range (*M* = 20.6; *SD* = 1.04) Six males


### Stimuli and Apparatus

Visual stimuli, which were presented on a 21-inch CRT monitor (100 Hz refresh rate), consisted of a fixation cross, a series of sequentially presented letters, and two disk figures. The white fixation cross (about 0.8° visual angle in size) was presented at the center of the monitor. The two black disk figures (both 1.5° in diameter) were presented 1.875° to the left and to the right of the fixation cross. The fixation cross and the two black disks were presented from the trial start to the end of the stimulus sequence presentation (3,780 ms).

#### Letter Sequence

**FIGURE 1:**
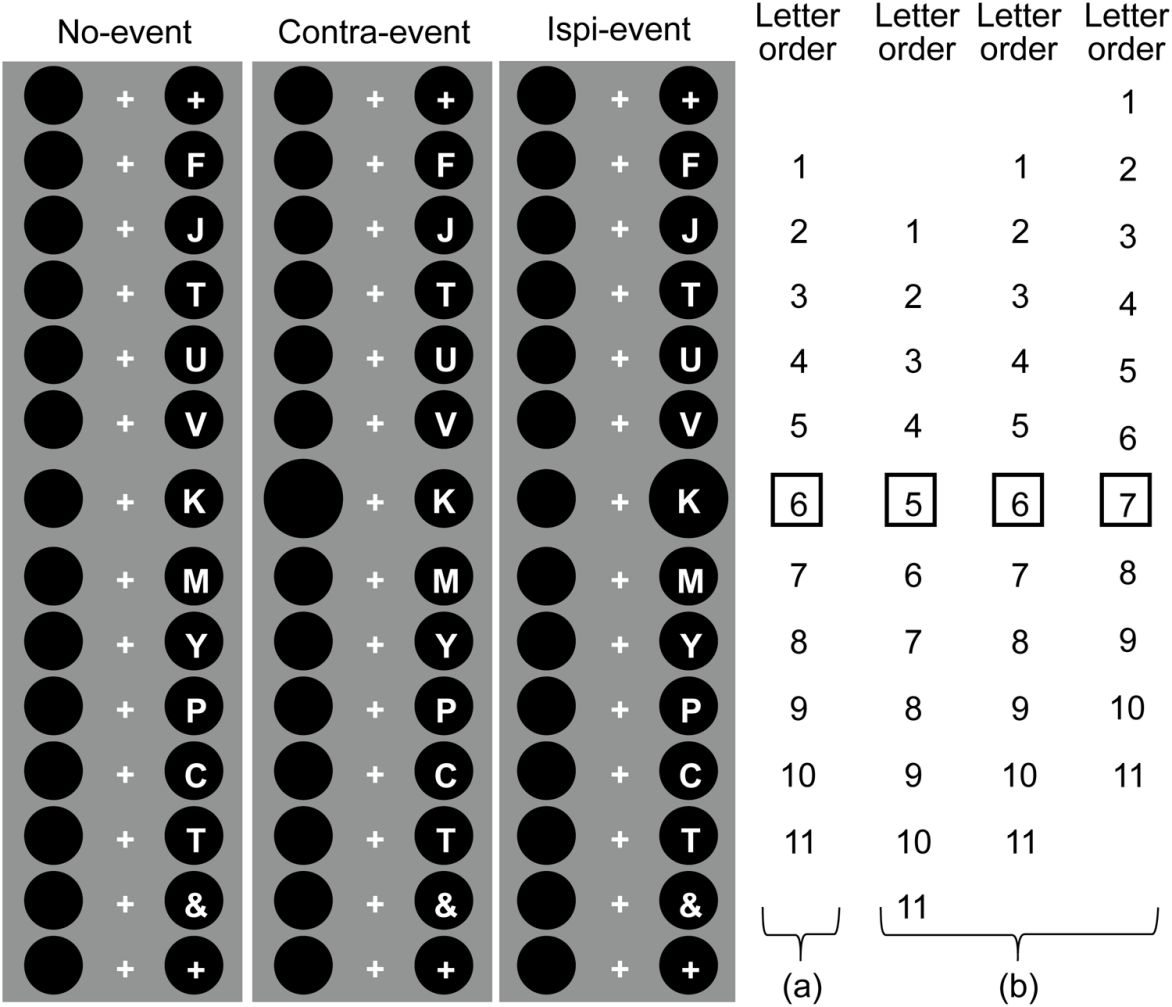
**Time course of the stimulus sequence and event occurrence (see Ohyama and Watanabe, 2010 for similar task illustration in a different design).** The vertical line in this figure represents the time course of the visual sequence. The event was a disk that enlarged simultaneously with the appearance of one letter in the serial sequence. The disk enlargement event occurred at the disk with the letter (ipsi-event) or at the disk on the other side (contra-event). In the spatially predictable condition, the ipsi- and no-event trials were shuffled in the ipsi-lateral event block, and the contra-vent and no-event trials were separately examined in the contra-lateral block. Thus, the event location was kept consistent in each block. In the spatially unpredictable condition, the ipsi-, contra-, and no-event trials were shuffled in a block; therefore, the location that the event occurred at was randomized. (a) In the temporally predictable condition, the event always occurred with the 6th letter in the sequence across all trials. (b) In the temporally unpredictable condition, the event occurred with the 5, 6, or 7th letter in the sequence, i.e., it varied across trials.

The serial stimuli sequence consisted of a series of 11 white letters (about 1.0° visual angle in size), located either 1.875° to the left or to the right of the fixation cross (see **Figure 1**). Thus, the letter sequence always appeared at the center of either side of the two black disks. Eleven letters were randomly selected from a set of 20 capital Roman letters (A, B, C, D, E, F, G, H, J, K, L, M, N, P, R, S, T, U, V, W). Following the letter sequence, an “&” symbol appeared and masked the eleventh letter. Each letter and the “&” appeared for 270 ms (3.7 letters per second) with no inter-stimulus interval, so that the total duration of the letter sequence was 3,240 ms.

#### Event Stimuli

In the serial letter sequence, one of the two black disks located in the background of the serial presentation of letters increased in size by 50% (2.25°) for 270 ms, and was simultaneously presented with one of the sequentially presented letters. We termed this expanded disk presentation a “task-irrelevant event” that occurred in the constant sequence of letters. This event occurred either on the same side of the disk as the serial letter sequence (ipsi-event condition), or on the other side of the letter sequence (contra-event condition), as shown in **Figure 1**.

### Procedure

Participants were seated 57 cm from the visual display in a room with dim, ambient light. Prior to the onset of the stimulus sequence, a “+” symbol appeared within one of the two (left or right side) black disks, indicating the position where the letters would appear. The presentation side of the serial letter sequence was manipulated in a blocked design so that it alternated every 12 trials. Each trial was initiated by a participant’s button press. The stimulus onset asynchrony of the letter sequence from the time of pressing the start button was 0, 270, or 540 ms, at which point the “+” was presented until the start of the letter sequence. After the letter sequence (11 letters and “&”), the “+” was again presented until 3,780 ms had elapsed from the trial start. The event appeared or did not appear during the stimulus sequence, and all participants were informed that the event was irrelevant to the task. Moreover, none of the participants in the four experiments were told whether the timing and location of the event would be kept constant or varied. The stimulus presentation design and instructions were identical for the working memory task and the target detection task.

#### Working Memory Task

Participants were instructed to memorize the 11 letters presented in the serial sequence. Immediately after the stimulus sequence appeared, two letters were presented at 1.875° to the left and right of the fixation cross. One of those letters was a target chosen from among the 11 letters in the preceding sequence. The other letter was a non-target letter that was chosen from among the remaining nine letters that had not appeared in the 11-letter sequence in that trial. Participants were asked to report which letter was presented in the preceding sequence by pressing the left or right keys on the computer keyboard. Memory performance for the target letters in five temporal positions was tested in different trials, including the target appearing simultaneously with the event, plus one or two targets appearing before and after the event.

#### Target Detection Task

The sequential presentation of the visual stimulus was identical to that in the working memory task; however, in the target detection task, participants were given one letter prior to the start of the stimulus sequence as a target. During the serial stimulus sequence, they were told to press an appropriate key as quickly as possible once they detected the target letter; thus, the RT for target detection was recorded.

#### Conditions and Trials

The design and conditions of trials were identical for both working memory and target detection tasks. There were three event conditions (ipsi-event, contra-event, and no-event), with 24 trials repeated for five temporal positions of a target letter (5 × 24 = 120 trials per event condition). In addition, six catch trials were randomly inserted for the other six targets in order to eliminate the possible use of the strategy of memorizing just five targets of interest [temporal position (6) × 4 repetitions = 24 trials per event condition]. Therefore, 144 trials (5 × 24 + 6 × 4) were completed for each condition. **Table 1** illustrates the temporal and spatial 2 × 2 event predictability design of the four experiments in the present study.

### Experiments

#### Experiment 1: Temporally and Spatially Predictable Events

Experiment 1 consisted of two blocks, one of which contained the ipsi- and no-event trials, and the other block contained the contra- and no-event trials. Participants were provided with the event location (ipsi- or contra-lateral) prior to the start of each block, so that the event location was predictable in both blocks. The onset time of the letter sequence was consistently delayed for 270 ms after the start button was pressed, and the event appeared between 1,620 to 1,890 ms thereafter, so that the event timing coincided with the sixth letter in the serial sequence. Therefore, the timing of the event was also predictable. The time course of the stimulus sequence, presented in a trial of both working memory and target detection tasks, is shown in **Figure 1**.

#### Experiment 2: Temporally Predictable but Spatially Unpredictable Events

In Experiment 2, the event timing was the same as in Experiment 1, i.e., it coincided with the sixth letter in the serial sequence. However, the ipsi-, contra-, and no-event trials were shuffled in one block so that the event randomly appeared at the ipsi-lateral side of the letter sequence, at the contra-lateral side, or did not appear. Therefore, the timing of the event was predictable, but the spatial location was unpredictable.

#### Experiment 3: Temporally Unpredictable but Spatially Predictable Events

In Experiment 3, the onset time of the letter sequence was randomly delayed after the start button was pressed (0, 270, or 540 ms). The event appeared between 1,620 to 1,890 ms from the button press so that it coincided with the fifth, sixth, or seventh letter (0, 270, or 540 ms delay from letter sequence onset, respectively; see **Figure 1**); therefore, the event timing could not be predicted solely from the order of letters. The relationship between the side that the event appeared on and the letter sequence was kept consistent through each of two ipsi- and contra-lateral blocks. Therefore, the spatial location of the event was predictable.

#### Experiment 4: Temporally and Spatially Unpredictable Events

In Experiment 4, the design of the event location was the same as in Experiment 2, and the design of event timing was the same as in Experiment 3. Therefore, the event was both temporally and spatially unpredictable.

### Statistical Analysis

For Experiments 1 and 3, there were four event conditions: two occurrence conditions (event and no-event) × two separate blocks of laterality of event location (ipsi and contra). Therefore, a repeated measures 4 × 5 analysis of variance (ANOVA) (four event conditions × five temporal positions of target: -2 to 2 in relation to the event) was performed on data from both the working memory and target detection tasks. For Experiments 2 and 4, three event conditions (ipsi-event, contra-event, no-event) were mixed in one block; therefore, a repeated measures 3 × 5 ANOVA (three event conditions × five target temporal positions) was performed. For all analyses, the effect size η^2^ was calculated. When necessary, Greenhouse–Geisser corrections were applied and original degrees of freedom together with Greeenhouse–Geisser ε are reported.

## Results

### Working Memory Task

**FIGURE 2:**
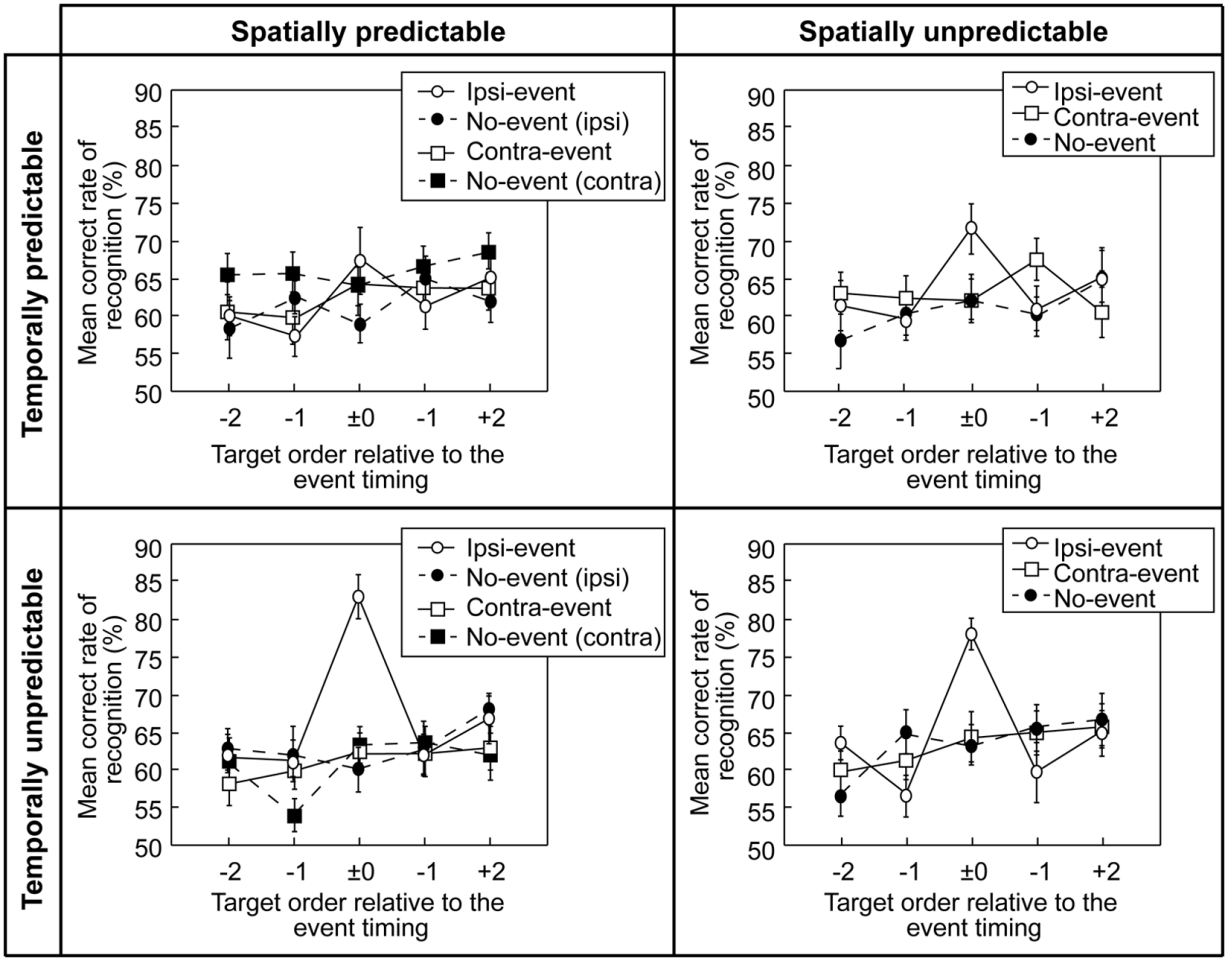
**Results of the working memory task in the four experiments.** Mean correct percentages (averaged over the participants) of short-term recognition memory performance are shown as a function of temporal position of the target letter relative to the event timing (designated as zero on the abscissa). Error bars denote the standard errors of the means. For the no-event condition, zero on the abscissa reflects the time of the letter presentation in the middle of a letter sequence (i.e., from 1,620 ms to 1,890 ms). Since recognition memory accuracy was based upon a response to one of two letters, the chance level was 50%. No-event (ipsi) is the no-event data in the ipsi-lateral event block, No-event (contra) is the no-event data in the contra-lateral event block.

**Figure 2** shows the mean correct percentages of recognition in the working memory task in the four experiments.

#### Experiment 1: Temporally and Spatially Predictable Events

In Experiment 1, the recognition memory performance was about 60% across all conditions, regardless of the different temporal positions. A repeated measures ANOVA indicated no significant differences in the main effect of the event condition, *F*(3,36) = 1.29, *p* = 0.294 [Greenhouse–Geisser correction, *ε* = 0.869, *F*(2.61,31.28), *p* = 0.295], η^2^ = 0.013, the relative temporal position, *F*(4,48) = 1.01, *p* = 0.412 [Greenhouse–Geisser correction, *ε* = 0.823, *F*(3.29,39.49), *p* = 0.404], η^2^ = 0.029, or the interaction between the two variables, *F*(12,144) = 1.12, *p* = 0.349 [Greenhouse–Geisser correction, *ε* = 0.543, *F*(6.51,78.16), *p* = 0.359], η^2^ = 0.058.

#### Experiment 2: Temporally Predictable but Spatially Unpredictable Events

In the no-event condition of Experiment 2, the recognition memory performance was about 60% across all conditions, regardless of the different temporal positions. In the ipsi-event condition, the recognition memory performance accuracy was slightly higher when the visual change occurred at the same time as the target letter presentation, but the difference was not significant. A repeated measures ANOVA indicated no significant difference in the main effect of the event condition, *F*(2,24) = 1.10, *p* = 0.349, η^2^ = 0.015, the relative temporal position, *F*(4,48) = 1.43, *p* = 0.239, η^2^ = 0.034, and the interaction between the two, *F*(8,96) = 1.61, *p* = 0.133, η^2^ = 0.069.

#### Experiment 3: Temporally Unpredictable but Spatially Predictable Events

In the no-event condition of Experiment 3, the recognition memory performance was about 60%, regardless of the different temporal positions. However, in the ipsi-event condition, the recognition memory performance accuracy was higher when the visual change occurred at the same time as the target letter presentation. In this case, the percentage of correct responses was close to 80%, indicating that the participants were better at recognizing the target that happened to coincide with the task-irrelevant event. A repeated measures ANOVA supported this observation, showing that there were significant main effects of the event condition, *F*(3,36) = 7.99, *p* = 0.000328, η^2^ = 0.075, and the relative temporal position, *F*(4,48) = 4.01, *p* = 0.00689, η^2^ = 0.058, and the interaction was also significant, *F*(12,144) = 3.21, *p* = 0.000417, η^2^ = 0.157. *Post hoc* Tukey’s honest significant differences (HSD) tests indicated that a significant difference in recognition memory performance between the letters that appeared simultaneously with the event at the ipsi-lateral side of the letters and the letters that were not coincident with events or letters in other conditions (i.e., the zero point on the *x*-axis of the ipsi-event condition in **Figure 2** was significantly higher than it was in the other target orders relative to the event of the ipsi-event condition, and was also significantly higher than all target orders in contra- and no-event conditions).

#### Experiment 4: Temporally and Spatially Unpredictable Events

In the ipsi-event condition of Experiment 4, the recognition memory performance accuracy was higher than the no-event and contra-event conditions when the visual change occurred at the same time as the target letter presentation. A repeated measures ANOVA indicated that although the main effect of the event condition was not significant, *F*(2,24) = 0.41, *p* = 0.67, η^2^ = 0.005, the main effect of the relative temporal position was significant, *F*(4,48) = 4.93, *p* = 0.0020665, η^2^ = 0.113. The interaction between the two variables was also significant, *F*(8,96) = 4.16, *p* = 0.000269, η^2^ = 0.167. *Post hoc* Tukey’s HSD tests indicated that the significant interactions were due to the significant increase in recognition memory of the letter coinciding with the event at the ipsi-lateral side, which is in line with the results of Experiment 3.

#### Cross-Experiment Analysis

We additionally did the direct comparison of the effect of ipsi-lateral event between experiments. Repeated measures mixed ANOVA indicated significant difference in the interaction between the temporal predictability (predictable, unpredictable) and timing (-2, -1, ±0, +1, +2), *F*(4,48) = 2.58, *p* = 0.0388, partial η^2^ = 0.13. *Post hoc* Ryan’s tests indicated that the significant difference between working memory performance in temporally predictable and unpredictable conditions at the expected event timing (the sixth letter in the sequence).

### Target Detection Task

**FIGURE 3:**
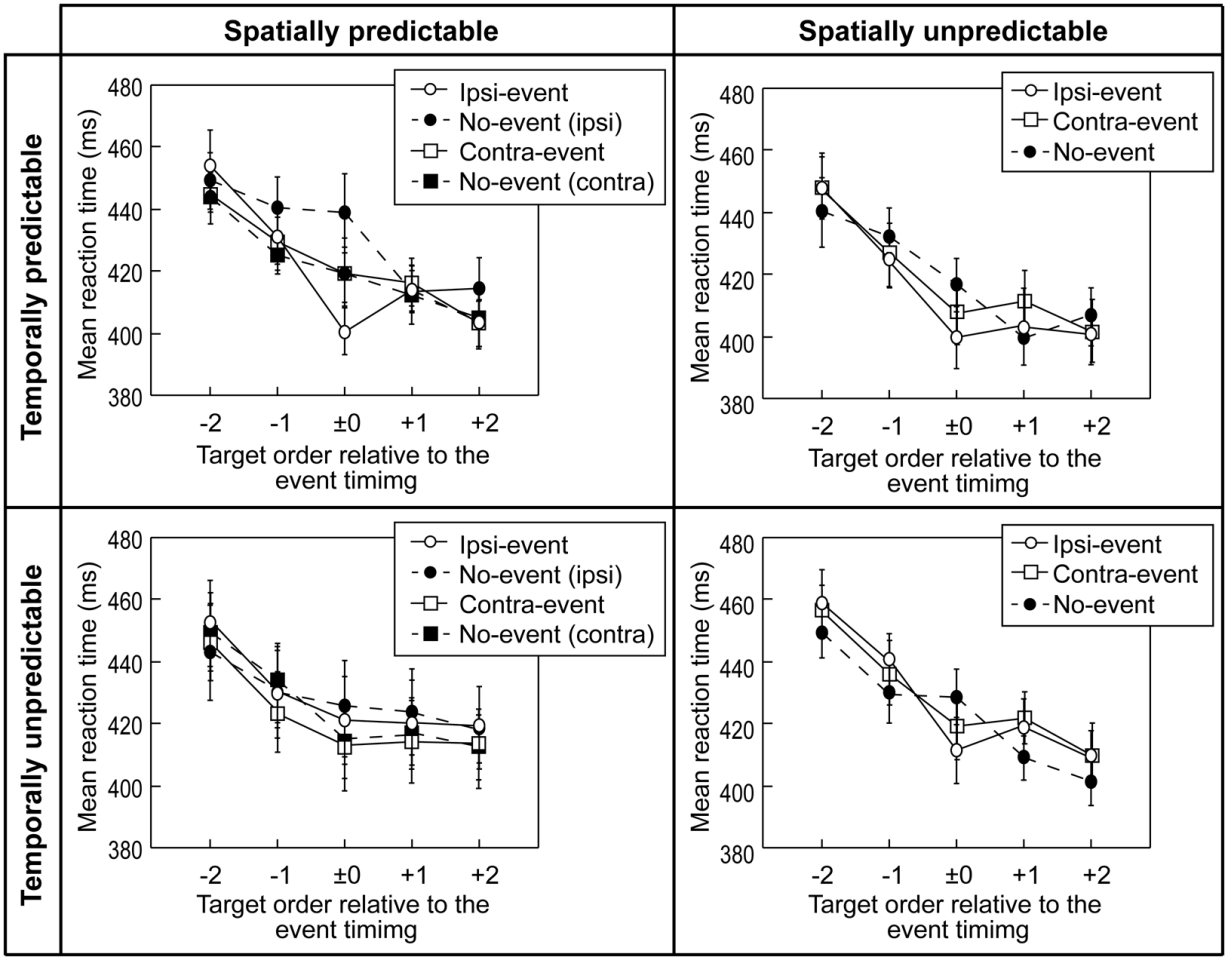
**Results of the target detection task in the four experiments.** Mean RTs (averaged over the participants) for correct detections are plotted as a function of the temporal position of the target letter relative to the event timing (designated as zero on the abscissa). Error bars denote the standard errors of the means. No-event (ipsi) is the no-event data in the ipsi-lateral event block, No-event (contra) is the no-event data in the contra-lateral event block.

**Figure 3** shows the mean RTs for the target detection task in the four experiments. RTs under 200 ms (which were regarded as a false start without detection) and over 810 ms (which were later than the offset time of the third letter from the target letter, and those that can be regarded as participants failing to detect the target) were excluded from the analysis; however, the exclusion rates were low (Experiment 1, 2, 3, and 4 were 3.8, 4.2, 2.9, and 3.6% respectively) and not significantly different across the four experiments.

#### Experiment 1: Temporally and Spatially Predictable Events

In Experiment 1, a repeated measures ANOVA indicated that the main effect of the event condition had little effect on the performance, *F*(3,36) = 2.12, *p* = 0.115, η^2^ = 0.032. The RTs in all the event conditions became shorter as target letters appeared later in the sequence, and the main effect of relative temporal position was significant, *F*(4,48) = 37.37, *p* < 0.0001, η^2^ = 0.357. In addition, the interaction was also significant, *F*(12,144) = 1.96, *p* = 0.031947, η^2^ = 0.053. *Post hoc* Tukey’s HSD tests indicated that the significant difference between detection performance coincided with the event at the ipsi-lateral side (ipsi-event condition) and detection performance of the no-event condition of the ipsi-lateral event block at the expected event timing (the sixth letter in the sequence).

#### Experiment 2: Temporally Predictable but Spatially Unpredictable Events

In Experiment 2, a repeated measures ANOVA indicated that the RTs in both ipsi- and contra-event conditions became shorter as target letters appeared later in the temporal positions, *F*(4,48) = 47.61, *p* < 0.0001, η^2^ = 0.488. However, the event condition had little effect on the performance, *F*(2,24) = 0.52, *p* = 0.6, η^2^ = 0.006, and the interaction was not significant, *F*(8,96) = 1.53, *p* = 0.155, η^2^ = 0.034.

#### Experiment 3: Temporally Unpredictable but Spatially Predictable Events

In Experiment 3, a repeated measures ANOVA indicated that RTs in both ipsi- and contra-event conditions became shorter as target letters appeared later in the temporal positions, *F*(4,48) = 38.69, *p* < 0.0001, η^2^ = 0.173. However, the event condition had little effect on the performance, *F*(3,36) = 0.26, *p* = 0.857, η^2^ = 0.013, and the interaction was not significant *F*(12,144) = 0.93, *p* = 0.522, η^2^ = 0.014.

#### Experiment 4: Temporally and Spatially Unpredictable Events

In Experiment 4, a repeated measures ANOVA indicated that the RT in both ipsi- and contra-event conditions became shorter as target letters appeared later in the temporal positions, *F*(4,48) = 38.93, *p* < 0.0001, η^2^ = 0.472. However, the event condition had little effect on the performance, *F*(2,24) = 0.97, *p* = 0.393, η^2^ = 0.01, and the interaction was not significant *F*(8,96) = 1.54, *p* = 0.155, η^2^ = 0.034.

## General Discussion

The results of Experiment 1 showed that when the occurrence of events was both temporally and spatially predictable, the events did not enhance working memory of sequentially presented items. However, with this dual predictability, when the events occurred in temporal conjunction with and at the same location as the target, the target detection speed was faster than it was for the target at the same timing but without the event. The results of Experiment 2 showed that when the occurrence of events was temporally predictable but spatially unpredictable, the events did not serve as cues for either memory or target detection processes. The results of Experiment 3 showed that when the occurrence of events was temporally unpredictable but spatially predictable, the visual change facilitated the memory process. The enhancement was limited to the event that occurred at the same location as the target in the letter sequence. In contrast, these events did not facilitate target detection. The results of Experiment 4 showed that when the occurrence of events was both temporally and spatially unpredictable, the events facilitated the memory process. Similar to the effect found in Experiment 3, the enhancement was limited to events that occurred at the same location as the target in a letter sequence. However, the temporally and spatially unpredictable events did not serve as cues for target detection. Additionally, in all four experiments, the RTs decreased for a target letter presented later in the sequence. This is a so-called *foreperiod effect*, whereby simple RTs decrease with an increase in interval duration before the appearance of the target (for a review, see Niemi and Näätänen, 1981). The effect was known as a general base line condition without cuing effect in the RT study. According to the post experiment subjective report, the participants did not notice that the location and timing of visual change was constant, regardless of whether the location or timing was constant or not. All the participants reported that they regarded the visual change was task-irrelevant and not related to the letter sequence task. These subjective reports were the same as the report in our previous study (Ohyama and Watanabe, 2010).

### Temporally Unpredictable (Surprising) Visual Events Evoke Bottom–Up Attention and Enhance Working Memory

One of the purposes of the present study was to examine how the spatial and temporal predictabilities of a visual event affect memory processes. A selective memory enhancement effect was found when a temporally unpredictable (i.e., surprising) event appeared at the same location as the target letter, irrespective of the spatial predictability of the event (Experiments 3 and 4).

Comparison of the findings of the four experiments in the present study suggested that temporal predictability played an important role in the memory enhancement effect, whereby memory accuracy was enhanced by visual change when an event was spatially coincident and temporally incongruent with the item sequence. In other words, the unexpected visual change seemed to act as a cue that attracted attention and activated the memory process. Interestingly, as the timing of the visual change became predictable, the effect on memory decreased. If the visual change is explicitly considered as a cue for activation of encoding or the memory process, a temporally predictable visual change should still have enhanced the short-term memory of a cued item. These findings imply that unexpected shifts in visual attention might cause the memory enhancement effect, which then disappears after the event timing is learned and becomes predictable. Memory enhancement seems to be linked to the unpredictable, surprising, or oddball-like properties of a visual event.

The results implied that the effect of event in condition of Experiment 2 was smaller compared to Experiments 3 and 4, but whether Experiment 2 had significant event effect or not remains unclear. There is a possibility that the common little spatio-temporal unpredictability effect underlies the results of Experiments 2, 3, and 4. However, it is difficult to explain the significant distinct effect in temporally unpredictable conditions in comparison with temporally predictable conditions. There could be an effect of any unpredictable event on memory, but it’s larger for temporal than spatial unpredictability.

This finding is partly consistent with previous studies of the effects of exogenous shift of visual attention on memory processes. These past studies have shown that some visual changes shifted attention to the spatial position of the change and affected visuospatial cognitive processes (Geng and Behrmann, 2002; Hollingworth and Henderson, 2002). Working memory accuracy of items at cued locations is selectively enhanced compared to items in other locations (Schmidt et al., 2002; Van der Stigchel et al., 2007). In contrast to the spatial cueing effect on memory, there have been few memory studies on the temporal cueing effect, except for those focused on the *serial position effect* (e.g., Murdock, 1962; Gershberg and Shimamura, 1994). Sequential memorization of items was influenced by salient cues (e.g., a target with a unique color and font, or with a peculiarly large font size), and it was more accurate than that of other less distinctive items in sequential memory, which is known as the von Restorff effect (von Restorff, 1933). Ohyama and Watanabe (2010) reported that the visual event affected only an item presented simultaneously with the event in a sequential presentation of items. The result of Experiment 3 in the present study was consistent with those of our previous reports, and showed that the memory enhancement effect was only evoked by the ipsi-event. The present findings are consistent with our previous report that task-irrelevant unpredictable visual changes enhanced working memory accuracy of an item simultaneously presented with an event that occurred within the same object in a stimulus sequence of items (Ohyama and Watanabe, 2010). Moreover, in the previous study, we smoothly connected two background disks into one dumbbell shaped object and found that contra-lateral events affect memory of the item simultaneously appeared with event. These findings provide further support for the idea that a visual cue affects working memory for an item only if the cue can be taken to be a part of the same item object, in accordance with an earlier finding that automatic perceptual grouping cues can bias the entry of items into the visual working memory (e.g., Woodman et al., 2003).

Moreover, previous studies have reported that recognition memory was enhanced when a surrounding scene was presented concurrently with a fixation target unrelated to the scene item in the time sequence of scenes (Lin et al., 2010; Swallow and Jiang, 2010). Their findings are similar to ours in that informative (defined by the degree of either task relevancy or surprise) events facilitate the memory process for surrounding co-occurring items at the event timing, although in our experiment, the event was irrelevant to the main memory task and the effect was confined to the location of the task. Therefore, the present results support the spatial and temporal selectivity of memory enhancement. Specifically, a surprising (i.e., temporally unpredictable) event may automatically attract bottom–up object-based attention to the timing and location of the event, which, in turn, might enhance the processes involved in recognition memory.

The present findings with regard to memory accuracy effects might be related to the long-term memory phenomenon of flashbulb memory, whereby an unexpected salient event is stored precisely and strongly in the long-term memory, along with other circumstantial information at that moment (Rubin and Wenzel, 1996; Talarico and Rubin, 2003). The cause of the flashbulb memory might be related to social or historical saliency and cannot be solely explained by the short-term recognition working memory advantage in the present study. However, the temporally unpredictable cueing effect might underlie the memory among sparse events within the countless parallel visual changes that occur in daily life.

### Temporally and Spatially Predictable Visual Events Drive the Learning Effect in Preparation for Top–Down Attention and Facilitate Detection

The RT results in the detection task indicated that changes in target detection speed did not correlate with changes in working memory. Visual changes facilitated detection RTs for the same side of the letter sequence only when the visual change was both spatially and temporally predictable. Further, if the events did not appear at the expected time and location, the prediction of the event seemed to slightly delay the target detection process. The advantage for detection RT also seemed to be limited to the target that appeared simultaneously with the event.

The significant influence of attentional cueing on visual target detection has been widely reported, whereby a visual cue that had previously appeared before items accelerated the detection speed of an item that appeared at the cued location compared with other items at an uncued location (Posner, 1980; Prinzmetal et al., 1986). For instance, Egly et al. (1994) demonstrated that the detection of a target at a cued location took the shortest time, but that the detection speed for a target at an uncued location in a cued object was faster than that for an uncued location in an uncued object. Moore et al. (1998) showed that when the location of a visual cue is congruent with a following target location (spatially valid cueing condition), the target detection speed is shorter than that for an uncued condition. Thus, a visual change might act as a cue to attract attention and, as such, have an after-effect on the perceptual processing of an item presented at the cued location or object. Under the condition of both spatially and temporally predictable events, as in Experiment 1, it is possible that the event was regarded as a cue, meaning that it is spatially valid and congruent with the target used in these previous studies.

The present results can be more consistently explained by endogenous (top–down), rather than bottom–up, attention, where prior knowledge on spatial locations of a target facilitates detection of the target (e.g., Posner, 1980; Theeuwes, 1991; Briand, 1998; Carrasco, 2011; Norman et al., 2013). Moreover, studies of the effect of temporal prediction of target onset on target detection speed have found reported that the detection RT of the target presented in expected timing was faster than that for the temporally unexpected target (Correa et al., 2004, 2005). Therefore, the enhancement effect of the detection RT in a spatially and temporally predictable event condition might be related to the previously reported effect of attention preparation.

We suggest that a task-irrelevant visual change occurring consistently at an expected time and location would passively and unconsciously be learned as a task-related valid cue, and bring attention to the expected timing and location. In this way, detection of an item that appears simultaneously with the visual change is facilitated. A temporally and spatially predictable event might be learned passively and implicitly regarded as a task-relevant valid cue, even though the event was task irrelevant. Therefore, the predictable event causes attention to be paid to the timing and location and accelerates the detection RT.

### Differential Effects of Temporal and Spatial Predictability on Detection and Memory

Does the effect of event on memory reflect the outcome of the effect on detection process, or is it a separate process? The present results clearly support the latter hypothesis. The RT was shorter in the temporally predictable conditions, while working memory, on the other hand, was enhanced when the event timing was unpredictable.

Similar discussions of the predictability of event occurrence, and how predictabilities of timing and location may affect the perceptual effect, have been reported in prior studies of subjective dilation of time perception. Tse et al. (2004) reported that the duration of an event was perceived as being longer for a temporally unpredictable and spatially predictable oddball event, in comparison with a standard non-salient event. By comparing empirically predictable and unpredictable conditions in a sequence, Pariyadath and Eagleman (2007) found that a temporally unpredictable event is an essential factor for subjective time perception change. This is consistent with the results of the present memory study, which vary depending on temporal predictability conditions. Therefore, it is possible that the duration of an item’s presentation with an event was perceived as being longer than the presentation duration of other items, and memory accuracy of the item with the event was improved. New and Scholl (2009) reported that a temporary and spatially unpredictable event evoked subjective time dilation of an item when the event position was different from that of the item. Their finding is consistent with the results of Experiment 2 in the present study, that spatial predictability does not affect the memory enhancement effect. However, they reported that subjective time expansion occurs even when the event happens to an object that is at a distance from the item. This result is not consistent with that in the present study, in that we did not find an effect on memory in the contra-lateral event condition, which is similar to the condition in the previous study. These results suggest that the subjective time expansion effect might occur for an item with a contra-lateral event; however, the memory enhancement effect was not driven by the item in the contra-event. Therefore, it is unlikely that the present findings can be explained solely by subjective time expansion. The memory enhancement effect in the present study, which was object-based, differs from the effect reported by New and Scholl (2009). However, temporal predictability in a sequence is a critical factor of memory enhancement effect, so the subjective time dilation model advocated by Pariyadath and Eagleman (2007) may be related to the memory enhancement effect. In brief, this indicates that the predictability of successive stimuli involves higher cortical areas than the primary visual cortex.

van Wassenhove et al. (2008) reported a similar effect within a spatially and temporally predictable event condition, observing that the contextual salience of stimuli is a critical factor and that unpredictable timing is just one of the factors related to this effect. Their experimental condition is similar to the condition of Experiment 1 in the present study, in which a temporally and spatially predictable event was used. Therefore, we consider that the subjective duration of a target item in the detection task of Experiment 1 in the present study was perceived as being longer than that of items without an event. This subjective time dilation might be related to the detection RT reduction, when the event was predictable in both time and space. Additionally, the previous study and our study indicate that if the event occurred consistently at the same timing and location through the blocks, the event timing and location were implicitly and automatically learned as task-relevant cues, attracted attention, and influenced the perception process, although the event was invalid and task-irrelevant.

It is possible that the participants used different strategies for memory and detection tasks. If the event attracts attention, increases arousal, and influences the early stages of the ongoing perception process, this automatic bottom–up effect would also have driven the early ongoing process in the working memory task, and would have a similar or larger memory enhancement effect in Experiment 1 than in Experiment 2. However, the memory enhancement effect was not seen in the event condition in Experiment 1, although the same condition of stimuli presentation had a significant effect on detection RT. Therefore, the present results suggest that unpredictable (or surprising) and predictable (or expected) events affect detection and memory processes differently. A convincing explanation for this difference has been suggested by Vogel et al. (2005), who found that a cue presented simultaneously with an item affected the memory but not perception of the target item at a cued location. They reasoned that the target item and the cue are processed in parallel and, therefore, the effect of the cue appeared only for post-perceptual processes, such as the memory of the items. We observed a similar effect on the working memory of items in a time sequence, which cannot be explained by the facilitation of the early detection process.

Moreover, it is reasonable to consider that the object-based attention underlies the effect of the event on memory and detection. Temporally unexpected event would attract attention and enhance memory of the item appeared simultaneously with the event. Our previous study and the results of contra-event conditions in the present study indicated that the memory enhancement effect was driven by an object-based attention. If a salient event occurred in another object from item sequence, the memory enhancement effect would not occurred irrelevantly to the spatial predictability. Moreover, we compared the memory enhancement effect in temporally predictable condition and unpredictable condition. If the event timing were predictable, the event would be regarded as a part of the stream and did not capture attention. Vice versa, for detection, an unpredictable event might distract from initial perception itself, and only a fully (i.e., both temporally and spatially) predictable event can aid target perception through expectation.

Zacks et al. (2007) proposed in their Event Segmentation Theory that the attentional gate is triggered by a change event and updates ongoing event perception. Swallow and Jiang (2010) reported a similar memory enhancement effect, whereby an unpredictable event at the center of view enhanced recognition memory of surrounding serial presentation of items (photographs of a natural scene), which is the spatially inversion of the condition used in our study. Their findings imply that unpredictable events enhance the memory process for a specific item that appears simultaneously with an unpredictable change, and support the perceptual prediction function in the event perception and memory processes. The present study also supported the perceptual prediction hypothesis by using clearly differentiated and controlled experimental conditions for assessing the spatio-temporal predictability of events. Other studies have reported that the learning effect of event timing affects the memory of a specific scene. For example, Ohyama and Watanabe (2007) reported that when a scene changes unpredictably, a flash that appeared before or after the scene change was memorized as if it appeared simultaneously with the change; however, this temporal memory averaging effect decreased when the change was predictable. These results indicate that the attentional gate for memory is not open when the timing is predictable, but when the timing is unpredictable, the attentional gate opens and has an impact on memory. In general, an unpredictable new change event might be regarded as the basis of a distinct memory of a salient impressive event compared with other, expected daily change events.

In addition, the present study is, to the best of our knowledge, the first report of the memory enhancement effect of a task-irrelevant surrounding event on serial presentation of items at the center of vision in a single task. In comparison, previous studies have reported that the memory enhancement effect was only found if participants paid attention to both the center event detection and surrounding item memory as a dual task, and that the effect disappeared in a single memory task. On the other hand, the results of the detection task implied that there was a positive learning effect of event prediction. The sequence of presentation of items might be perceived by predicting an event’s timing and location, although the event is task irrelevant. If a change event is predictable, top–down attention is focused on predictable change, and this predictable change can be precisely perceived as a known change event in the ongoing perceptual process. Furthermore, the present study showed that a learned predictable event does not affect recognition memory. In general, the situation is easily perceived in an accustomed context; however, saliency or peculiarity is low when the event is well-known and expected. Thus, the learning effect of predictability may underlie our observation that the memory enhancement effect was not evoked by a temporally predictable event.

Presenting events that are expected in terms of both location and timing facilitates detection but not memory processes. One possible explanation for this is that the learning effect of the occurrence of a visual event might drive predictive top–down attention and enhance the ability to detect an item at the expected location and timing. From the point of view of memory processes, on the other hand, the expected change in sequence would be less informative; therefore, surprising shifts in visual attention in the memory process might be restrained under predictive conditions.

It remains to be seen whether the present results are generalizable to most situations. For example, if the cue is more salient and/or attention grabbing, even an item presented with a predictable cue might enhance the memory, as per the von Restorff effect. Further studies are needed to examine how temporal and spatial predictabilities of visual events interact with other properties of the cue (such as saliency and task-relevancy) and dynamics of attention shifts.

## Author Contributions

JO: Designed and performed the experiments, analysis, and wrote the manuscript. KW: Contributed to all parts of this study.

## Conflict of Interest Statement

The authors declare that the research was conducted in the absence of any commercial or financial relationships that could be construed as a potential conflict of interest.
